# Comparing Osteoarthritis Burden Across Central, Eastern, and Western Europe (1990-2021): Insights From the Global Burden of Disease Study 2021

**DOI:** 10.7759/cureus.93585

**Published:** 2025-09-30

**Authors:** Kenechukwu Igbokwe, Kenneth Ugwoke, Obak L Obak, Aminu Igwe, Jethro M Ngwu

**Affiliations:** 1 Trauma and Orthopedics, Gateshead Health NHS Foundation Trust, Gateshead, GBR; 2 Vascular Surgery, United Lincolnshire Hospitals NHS Trust, Gonerby Hill Foot, GBR; 3 Trauma and Orthopedics, Aberdeen Royal Infirmary and Woodend Hospital, NHS Grampian, Aberdeen, GBR; 4 General Practice, Hull University Teaching Hospital, Hull, GBR; 5 General Practice, Worcestershire Royal Hospital, Worcestershire Acute Hospitals NHS Trust, Worcester, GBR

**Keywords:** disability-adjusted life year, global burden of disease (gbd), high body mass index, under 50 years aged, years lost to disability

## Abstract

Background and aim

Osteoarthritis ranks as one of the leading causes of disability, predominantly affecting adults aged 70 years and older. This study aimed to report trends in the burden of osteoarthritis from 1990 to 2021 based on the Global Burden of Diseases Study (GBD) 2021 across Central, Eastern, and Western Europe.

Methods

This study is a systematic analysis of secondary database estimates from the GBD 2021 study for osteoarthritis in Central, Eastern, and Western Europe, as well as the 44 countries. The age-standardized rates of incidence, prevalence, and years of life lost due to disability were evaluated by age, gender, and high body mass index (BMI).

Results

In 2021, there were 101.2 million (95% uncertainty interval {UI}, 90.4-112.6 million) prevalent cases of osteoarthritis, a 48.3% increase from 1990. Subregional estimates showed that the incidence, prevalence, and years lived with disability (YLDs) cases in Western Europe were more than twice the number in Eastern Europe and more than four times the number in Central Europe, and had higher disability rates in young adults than their two other counterparts. Osteoarthritis accounted for approximately 6.7 million (95% UI 4.1-5.2 million) incidents, and there were 3.6 million YLDs (95% UI, 1.7-7.3) in Europe across all age groups. There was a positive association between the rising BMI in individuals under 50 years of age and the age-standardized rates of YLDs.

Conclusion

Between 1990 and 2021, the prevalence of osteoarthritis across Europe has consistently increased across all age groups, exerting considerable pressure on the workforce as it increasingly affects individuals under 50 years of age. To help reduce this impact, the World Health Organization advocates recognizing high BMI as an independent disease, an approach that would directly address a key contributor to the heightened disability and health complications linked to osteoarthritis.

## Introduction

Osteoarthritis is a complex disease characterized by inflammatory and metabolic factors, marked by the progressive breakdown of articular cartilage and changes in surrounding bone and soft tissues, commonly affecting the knees, hips, hands, and spine [1,2]. It is among the leading causes of pain, disability, and reduced quality of life in older adults globally [2,3]. According to the Global Burden of Disease (GBD) 2021 study, osteoarthritis accounted for a substantial portion of years lived with disability (YLDs) worldwide, ranking as the seventh highest cause of YLDs in adults aged 70 years and older, reflecting its high prevalence and chronic disabling nature [3,4].

The Global Burden of Disease (GBD) study is an ongoing effort aimed at quantifying health outcomes worldwide. The GBD 2021 study incorporated new data sources and enhanced analytical techniques to produce revised yearly estimates of mortality, life expectancy, and population figures across global, regional, national, and subnational scales from 1950 through 2021 [5]. It has shown rising incidence and prevalence of osteoarthritis globally, positively correlating with the sociodemographic index (SDI) following a north-south gradient [6,7]. Higher incidence and prevalence were reported in areas with higher sociodemographic index and vice versa [8]. These variations have been attributed to the burden of high body-mass index (BMI), aging, and the availability of health infrastructure. However, as with many non-communicable diseases, the burden of osteoarthritis is not uniformly distributed across Europe due to established variations in population dynamics in Eastern, Central, and Western Europe [6].

Understanding regional differences in the burden of osteoarthritis is essential for planning effective prevention and management strategies, as well as for informing future priorities and identifying avoidable health disparities associated with modifiable risk factors. Therefore, the objectives of this study were to (1) compare the GBD 2021 estimates of osteoarthritis-related indices - incidence, prevalence, and years lived with disability (YLDs) - across countries in Central, Eastern, and Western Europe; and (2) examine temporal trends in osteoarthritis-related YLDs among individuals under the age of 50 years between 1990 and 2021 by region. This will be achieved by presenting both absolute counts and age-standardized rates, which will effectively disentangle the impact of population growth on the evolving burden of osteoarthritis.

## Materials and methods

Data source

The GBD 2021 study, conducted by the Institute for Health Metrics and Evaluation (IHME), integrates all existing data to provide consistent information on the burden of diseases, injuries, risk factors, and the latest global, regional, and national estimates. For the current study, we utilized data from GBD 2021, a more detailed description of which has been previously published [5].

Study design

This study is a secondary database systematic analysis of GBD 2021 study estimates for osteoarthritis in Central, Eastern, and Western Europe, and the 44 countries, i.e., in the Central sub-region - Albania, Bosnia and Herzegovina, Bulgaria, Croatia, Czech Republic, Hungary, Montenegro, North Macedonia, Poland, Romania, Serbia, Slovak Republic, and Slovenia; in the Eastern sub-region - Belarus, Estonia, Latvia, Lithuania, Moldova, Russian Federation, and Ukraine; and in the Western sub-region - Andorra, Austria, Belgium, Cyprus, Denmark, Finland, France, Germany, Greece, Iceland, Ireland, Israel, Italy, Luxembourg, Malta, Monaco, Netherlands, Norway, Portugal, San Marino, Spain, Sweden, Switzerland, and the United Kingdom.

In GBD 2021, illnesses and injuries were coded using the International Classification of Diseases, Ninth Revision (ICD-9) and the International Statistical Classification of Diseases and Related Health Problems, 10th Revision (ICD-10) to create a comprehensive hierarchy of nested levels. The GBD's four-level standard hierarchical categories were used to group the cause-of-injury categories. Level 1 causes fall under the "non-communicable diseases" category (Group II). Level 2 causes fall under the "musculoskeletal disorders" group. Level 3 causes fall under the "osteoarthritis" group. The data obtained from the GBD 2021 for osteoarthritis included the prevalence, incidence, and years lived with disability (YLDs) of the three regions [9]. We have used YLDs (instead of disability adjusted life years, DALYs) to quantify the burden of disease, given that the cause-of-death model in GBD estimation does not assume a causal relationship between osteoarthritis and mortality.

Osteoarthritis cases were identified using the Global Burden of Disease (GBD) 2021 criteria, which define the condition as symptomatic osteoarthritis in the hip, knee, hand, or other joints, confirmed by radiographic evidence. Radiographic diagnosis followed the Kellgren-Lawrence grading system, with eligible cases classified as grade 2 (definite osteophytes and joint space narrowing) or grades 3-4 (multiple moderate osteophytes, clear joint space narrowing, subchondral sclerosis, and joint deformity). To meet the case definition, individuals also had to report joint pain lasting at least one month within the previous 12 months. This combined clinical and radiographic approach ensured consistent identification of symptomatic osteoarthritis across datasets. In GBD 2021, some estimates showed a negative impact, meaning the lower end of the 95% uncertainty interval was less than one, because the analysis included differences between studies when calculating relative risks. This means the results sometimes suggested no link, or even a possible protective effect, between the risk factor and the health outcome. When this happens, the wide uncertainty range typically indicates a weak or unclear relationship. To be transparent, we show the full range of uncertainty in our results.

Data processing

Although the Global Burden of Disease (GBD) 2021 study provides osteoarthritis data spanning from 1990 to 2021, we used the R programming language to create visual representations of prevalence, incidence, and years lived with disability (YLDs) both overall and stratified by age, sex, and risk factor, using publicly available data from the Institute for Health Metrics and Evaluation website (https://vizhub.healthdata.org/gbd-results/) [10]. The percentage change from 1990 to 2021 was determined by subtracting the 1990 estimate from the 2021 estimate and dividing the result by the 1990 value. A positive value reflects an increase in disease burden over the study period, while a negative value indicates a reduction. Estimates of the number of individuals living with osteoarthritis and age-standardized prevalence were presented with 95% uncertainty intervals (UIs), and all rates were expressed per 100,000 population.

## Results

This study revealed that there were about 101.2 million (95% UI, 90.4-112.6 million) prevalent cases of osteoarthritis, with an age-standardized prevalence estimate of 39326.4 per 100,000 people (95% UI, 34907.6.7-43879.3). This was a 48.3% increase from 1990 to 2021. In addition, osteoarthritis accounted for approximately 6.7 million (95% UI, 4.1-5.2 million) incident cases in the region, with an age-standardized incidence rate of 2615.5 (95% UI, 2316.7-2921.6), representing a 37.4% increase between 1990 and 2021. In 2021, there were 3.6 million YLDs (95% UI, 1.7-7.3) in Europe across all age groups, with an age-standardized rate of 466.1 (95% UI, 227.8-942.9) YLDs per 100,000 population. This represents a 49.9% increase between 1990 and 2021.

At the subregional level, the incidence counts of osteoarthritis in 2021 were highest in Western Europe (39,189 {95% UI, 34,984-43,632}) and lowest in Central Europe (96.01 {95% UI, 8494-10,703}). In 2021, the age-standardized incidence rate was also highest in Western Europe and lowest in Central Europe. Central Europe experienced the largest change in rates from 1990 to 2021, with a 49.0% increase. The 2021 age-standardized prevalence per 100,000 people with osteoarthritis appeared to have minute differences in the distribution with rates of 12,597 per 100,000 population (95% UI, 11,100-14,084), 13,111 per 100,000 population (95% UI, 11,496-14,715), and 13,618 per 100,000 population (95% UI, 12,311-15,080) in Central, Eastern, and Western Europe, respectively (Table 1).

**Table 1:** Number and age-standardized rates of incidence, prevalence, and YLDs in 1990 and 2021 for regional osteoarthritis, and percentage change from 1990 for each measure in Central, Eastern, and Western Europe. UI: uncertainty interval; YLDs: years lost to disability

Variables	Central (95% UI)	Eastern Europe (95% UI)	Western Europe (95% UI)
Incidence counts (×10^4^)	96.01 (84.94-107.03)	183.30 (161.25-205.82)	391.89 (349.84-436.32)
Age-standardized incidence rates, 1990 (×10^2^)	5.59 (4.94-6.23)	6.60 (5.79-7.43)	6.84 (6.11-7.62)
Age-standardized incidence rates, 2021 (×10^2^)	8.33 (7.37-9.29)	8.90 (7.80-9.95)	8.96 (8.00-9.98)
Percentage change in age-standardized rates between 1990 and 2021 (%)	49.01	34.3	30.92
Prevalence (×10^4^)	1451.94 (1279.43-1623.42)	2710.79 (2376.93-3042.36)	5956.74 (5384.90-6596.00)
Age-standardized prevalence rates, 1990 (×10^2^)	74.98 (66.15-84.02)	92.92 (81.45-104.85)	97.21 (87.53-107.48)
Age-standardized prevalence rates, 2021 (×10^2^)	125.97 (111.00-140.84)	131.11 (114.96-147.15)	136.18 (123.11-150.80)
Percentage change in age-standardized rates between 1990 and 2021 (%)	67.99	41.09	40.09
Years lived with disability (YLDs) (×10^4^)	51.44 (24.81- 104.43)	96.56 (46.51-195.17)	213.13 (103.84-428.73)
Age-standardized YLD rates, 1990 (×10^2^)	2.61 (1.26-5.27)	3.28 (1.57-6.66)	3.45 (1.67-6.94)
Age-standardized YLD rates, 2021 (×10^2^)	4.46 (2.15-9.06)	4.67 (2.24-9.43)	4.87 (2.37-9.80)
Percentage change in age-standardized rates between 1990 and 2021 (%)	70.74	42.3	41.12

**Figure 1:**
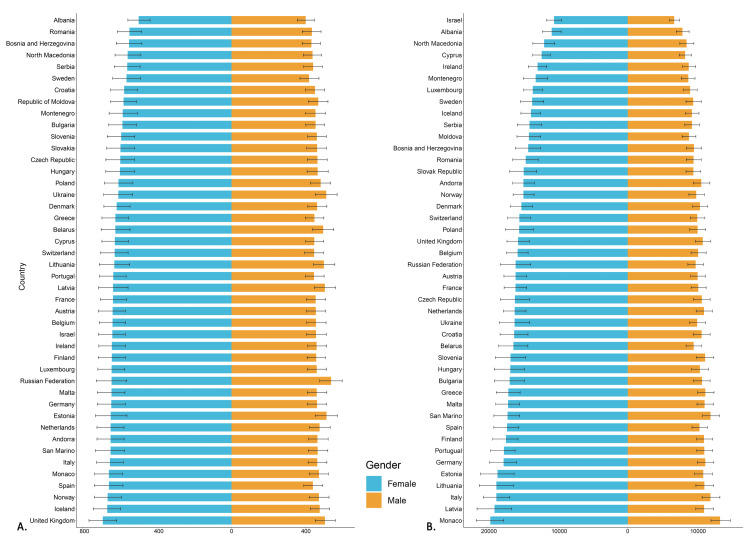
Male and female age-standardized rates for all European countries, with error bars showing uncertainty intervals. (A) ASR of incidence for osteoarthritis in 2021. (B) ASR of prevalence for osteoarthritis in 2021. ASR: age-standardized rate

Country-level data showed the highest age-standardized incidence rates of osteoarthritis in the United Kingdom (599.09 {535.27-664.80}) and the Russian Federation (595.22 {523.11-664.62}). While Monaco (32716.4 {95% UI, 36173.1-29511.8}), Italy (30488.1 {95% UI, 33691.4-27364.4}) and Latvia (29842.9 {95% UI, 33657.5-26201.2}) had the highest osteoarthritis age-standardized prevalence rates (ASPR) in 2021, the ASPR were lowest in Albania (18618.6 {95% UI, 20874.4-16398.7}) and North Macedonia (17119.3 {95% UI, 18915.4-15402.7}) (Figure 1). The country-level age-standardized prevalence and incidence estimates for all European countries, presented by gender, are shown in Figure 1.

Age patterns

**Figure 2:**
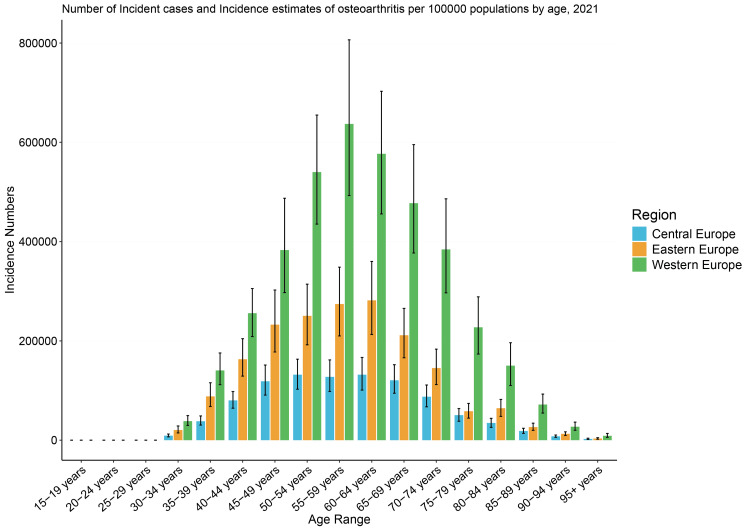
Incident case counts and incidence estimates per 100,000 population by age in 2021, with error bars representing uncertainty intervals.

Analysis by age group revealed distinct patterns in the burden of osteoarthritis. Although individuals aged 55-59 years accounted for the most incident cases in the western subregion, the highest age-standardized incidence rates (ASIRs) were observed among individuals aged 60-64 years in Western Europe (2077.48 {95% UI, 1641.41-2531.10}) (Figure 2). Individuals aged 55-59 years had the highest ASIR in Central (1796.58 {95% UI, 1382.07-2277.76}) and Eastern Europe (1993.69 {95% UI, 1526.73-2533.66}) (Table 2). The age-standardized rates of prevalent cases increased with age up to the oldest group (95+ years). Sub-regional estimates revealed the highest prevalence rates at varying ages, peaking at 60-64 years in the Eastern Region, 65-69 years in the Central Region, and 70-74 years in the Western Region (Table 2). Individuals aged under 50 years accounted for approximately 16% of ASIRs in all three regions (Table 2).

**Table 2:** Age-standardized incidence and prevalence rates for osteoarthritis in Central, Eastern, and Western Europe by age group, based on 2021 data from the Global Burden of Disease Database study.

Variables	Central Europe (95% UI)		Eastern Europe (95% UI)		Western Europe (95% UI)	
Age group	Incidence	Prevalence	Incidence	Prevalence	Incidence	Prevalence
15-29 years	0	0	0	0	0	0
30-34 years	116.08 (85.42-157.44)	259.68 (196.87-333.46)	124.25 (88.41-173.68)	279.74 (208.49-362.68)	138.78 (107.52-179.08)	318.29 (252.89- 398.97)
35-39 years	447.92 (357.49-573.60)	1551.69 (1229.00-1983.04)	502.50 (386.84-658.88)	1702.015 (1329.071-2164.362)	499.34 (397.16-624.20)	1795.56 (1457.64-2223.32)
40-44 years	891.16 (716.31-1089.31)	4570.05 (3755.85-5609.31)	1046.97 (828.94-1313.18)	5208.529 (4229.344-6480.278)	883.04 (720.69-1054.47)	4856.42 (4027.85-5819.59)
45-49 years	1368.42 (1047.89-1746.05)	9446.25 (7836.77- 11273.69)	1607.62 (1226.93-2088.88)	11023.22 (9050.80-13308.28)	1286.60 (998.66-1637.20)	9323.23 (7794.22-10927.32)
50-54 years	1682.28 (1309.32-2080.37)	15560.05 (13088.87- 18529.65)	1925.08 (1476.92-2415.83)	18222.98 (15280.82-21825.19)	1707.65 (1376.38-2070.85)	15089.57 (12881.22-17733.82)
55-59 years	1796.58 (1382.07-2277.76)	21975.45 (18511.28- 25420.97)	1993.69 (1526.73-2533.66)	25370.04 (21279.79-29574.46)	2029.77 (1570.07-2569.45)	21743.61 (18680.72-24809.22)
60-64 years	1735.34 (1327.49-2191.63)	27692.61 (23602.92-31590.27)	1894.89 (1432.45-2422.17)	31613.8 (26845.21-36311.09)	2077.48 (1641.41-2531.10)	28334.41 (24615.39-32214.16)
65-69 years	1612.69 (1264.82-2032.54)	32414.85 (28231.68-37075.07)	1747.38 (1372.52-2194.94)	36688.15 (31785.59-42171.33)	1974.40 (1559.35-2462.14)	33971.25 (29916.99-38126.94)
70-74 years	1470.88 (1128.76-1867.41)	36242.98 (31864.85- 40929.66)	1579.92 (1218.50-1994.99)	40725.42 (35589.85-46164.02)	1724.54 (1332.07-2182.07)	38548.12 (34086.35-43142.34)
75-79 years	1329.42 (1004.30-1676.45)	39062.21 (34643.83- 43727.91)	1416.67 (1078.67-1798.90)	43665.97 (38577.38-48914.03)	1377.86 (1051.07-1749.55)	41408.83 (37125.87-45979.38)
80-84 years	1254.98 (934.16-1589.79)	41457.51 (36792.98- 46065.74)	1337.28 (995.82-1705.29)	46294.17 (41097.01-51544.77)	1099.55 (806.43-1437.58)	42969.78 (39046.28-47391.08)
85-89 years	1193.87 (889.29-1548.70)	43666.18 (38742.47- 48336.68)	1268.34 (949.22-1641.25)	48171.11 (42728.01-53313.08)	849.25 (645.57-1097.76)	43534.07 (39431.95-47961.57)
90-94 years	1301.57 (959.88-1721.55)	45846.20 (40799.53- 50808.11)	1368.47 (1018.51-1802.88)	50149.81 (44540.23-55381.11)	726.49 (534.63-974.47)	43601.18 (39420.67-47935.55)
95+ years	1585.08 (964.78-2445.03)	48600.52 (43335.79- 54113.76)	1652.45 (1009.79-2519.16)	52929.6 (47219.69-58501.93)	760.29 (497.12-1180.29)	43893.59 (39625.52-48707.90)

Individuals aged between 15 and 49 years contributed 304,000 YLDs (95% UI, 139924.41-623753.40). For this age group, the age-standardized YLDs increased over the three decades from 1990 to 2021, increasing by 53.8% in Central Europe, 44.3% in Eastern Europe, and 33.6% in Western Europe. Sub-regional estimates showed a significant increase in age-standardized YLDs contributed by individuals under 50 years of age to the overall age-standardized rates in the three regions. In 1990, individuals under 50 years contributed 26.7%, 25.4%, and 34.7% of the overall YLD rate in Central, Eastern, and Western Europe, respectively. However, in 2021, the contributions of YLDs in individuals aged under 50 years to the overall age-standardized YLD rates for Central, Eastern, and Western Europe were 36.6%, 34.7%, and 34.1%, respectively, demonstrating the increasing burden of disability in this population. At both times, Eastern Europe had the highest disability-related osteoarthritis burden among individuals aged under 50 years in Europe (Table 3). Age-standardized YLD rates are distributed by age groups in the three European subregions as shown in Table 3.

**Table 3:** Age-standardized YLDs rates and high BMI age-standardized YLDs rates distributed by age groups in the three European subregions. YLDs: years lost to disability

Variables	1990		2021	
	15-49 years	Age-standardized	15-49 years	Age-standardized
Central Europe	58.33 (26.58-119.27)	218.64 (104.72-441.82)	89.72 (40.74-185.08)	245.41 (117.63-496.21)
Eastern Europe	67.46 (30.34-139.73)	265.83 (126.81-540.87)	97.37 (44.32-201.48)	280.78 (134.37-567.03)
Western Europe	64.74 (29.92-131.70)	238.56 (115.19-479.88)	86.47 (40.22-176.31)	253.58 (123.06-510.55)
Age-standardized YLD rates attributable to high BMI				
Central Europe	11.14 (-1.02-29.15)	42.05 (-3.89-114.92)	18.65 (-1.87-48.23)	51.25 (-5.32-138.21)
Eastern Europe	10.11 (-0.89-26.99)	42.78 (-3.96-118.19)	18.35 (-1.92-47.28)	54.95 (-5.69-144.57)
Western Europe	13.32 (-1.21-34.95)	50.71 (-4.69-140.96)	21.39 (-2.13-54.98)	62.95 (-6.27-168.56)

Attributable risk factors for the osteoarthritis burden

**Figure 3:**
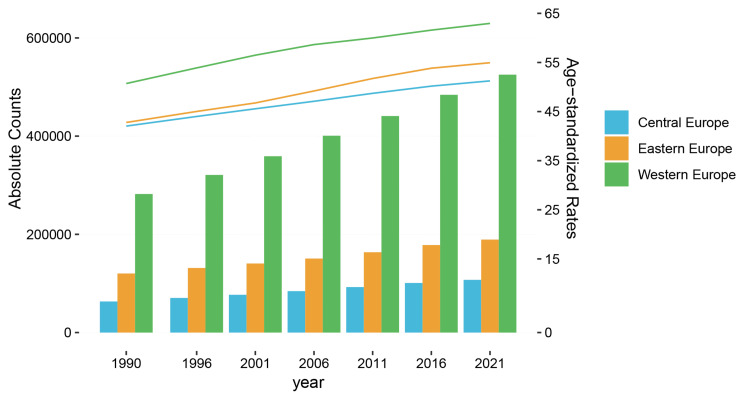
Absolute counts and age-standardized YLDs attributable to osteoarthritis in Central, Eastern, and Western Europe from 1990 to 2021. YLDs: years lived with disability

In 2021, high body mass index (BMI) was the only GBD risk factor for osteoarthritis, and it accounted for over 821,000 osteoarthritis YLDs (821808.21 {95% UI, -82123.1944-2202570.92}) in the European regions, with a 76.5% increase from 1990 (465672.83 {95%UI, -43001.66-1290185.68}). The contribution of high BMI to age-standardized YLDs of osteoarthritis increased over time, with 21.9%, 28.5%, and 24.1% increases in Central, Eastern, and Western Europe, respectively, between 1990 and 2021 (Figure 3). Individuals under 50 years of age had significantly increased YLDs attributable to osteoarthritis, increasing by 67.5%, 81.5%, and 60.5% in Central, Eastern, and Western Europe, respectively (Table 3). Despite these significant differences over 30 years, Western Europe had the highest disability attributable to high BMI for osteoarthritis in individuals aged under 50 years and in the regional population.

## Discussion

The global burden of osteoarthritis has been reported to be increasing in several articles, correlating positively with SDI in a north-south gradient [4,11]. In 2020, it was a top 10 leading cause of disability in people aged above 70 years [4]. Between 1990 and 2021, there was an overall increase (48.3%) in the age-standardized incidence rates in Europe. Although global estimates showed an increase in the disability counts (YLDs) with a relatively stable age-standardized rate, regional estimates within Europe showed significant increments in age-standardized rates, which were highest in central Europe over the three decades (49.0%) [4]. In comparison to all three regions, absolute counts of incidence, prevalence, and YLDs were highest in Western Europe, being more than twice the number in Eastern Europe and more than four times the number in Central Europe. These significant differences could be attributed to population differences between the three regions, given the relatively comparable age-standardized rates of these indices per 100,000 population.

Country-level estimates revealed a high incidence of osteoarthritis in females, a finding consistent across the European region and reported in other studies, accounting for approximately 60% of people with osteoarthritis globally [12-15]. The increased incidence in females is a result of significant differences in biomechanical analyses of joint force components in men and women [16]. These biomechanical differences have been established in the knee and explain the increased susceptibility of lower limb injuries in women when the load (and/or BMI) increases [17]. Anatomical differences between men and women, such as a wider pelvis and greater quadriceps angle, may confer greater varus-valgus laxity [18] and malalignment that could contribute to the higher rate of knee injuries (five-fold rate of anterior cruciate ligament tears) and further increase the risk of developing osteoarthritis later in life [17,19]. Given that estrogen is protective against cartilage degradation, post-menopausal changes in estrogen levels could potentially increase the risk of incident osteoarthritis but this is not conclusive [20].

This study found that although the prevalence of osteoarthritis increased with each decade of life, the prevalence peaked earliest in Eastern Europe between 60 and 69 years and latest in the early 70s (70-74 years) in Western Europe. Studies in the United States and China have shown an increasing prevalence of knee osteoarthritis each decade, with a significant surge in incidence after the age of 55 [12,13]. The incidence was highest in the three European subregions among individuals aged 55-64 years. This peak in incidence has been believed to be due to differences in the cellular response to inflammation within the articular cartilage at these ages [13]. Cellular responses included secretion of less synovial fluid, reduced cytokines and growth factors activity in response to injury, and reduced muscle strength to support joint function, which leads to accelerated cartilage wear and degeneration [13].

A population-based study in England found that the incidence of osteoarthritis increased significantly between 2003 and 2010 in individuals aged 35-44 years, per 1,000 population [21]. Beyond this data, there is no directly reported estimate of disability attributable to osteoarthritis in individuals under 50 years; however, several reports have stated an increasing incidence in working-age people globally [6]. In this study, individuals under 50 years of age accounted for approximately 16% of the overall incidence of osteoarthritis in the European sub-regions. Although this is lower than the 19.2% incidence in India, it implies that approximately one in six people diagnosed with osteoarthritis in Europe is under 50 years, and this will present a significant impact on the labor force as it affects a significant proportion of the working-age population in the region [22]. The rising age-standardized YLD rates in young people in Europe have also been reported in global studies showing the progressive growth [5]. Although there is insufficient evidence to explain why osteoarthritis is rising in young people, it is possible that the increased self-awareness of healthcare problems and increased diagnostic capacity may have led to increased incidence and overall disability.

A high BMI is defined as a value of ≥25 kg/m^2^ and is a significant risk factor affecting the incidence of osteoarthritis globally. It is primarily the result of combined modifiable risk factors such as poor behavioral risk factors, including sedentary lifestyles, unhealthy diet, and lack of physical activity. Global adult BMI has shown a sustained upward trend since 1975, with increasing prevalence in most countries [14]. The percentage of YLDs attributed to high BMI has been reported to have increased significantly by 4.5% globally between 1990 and 2021 [23]. Given that obesity in countries with high sociodemographic index has increased significantly between 1990 and 2021 in individuals aged under 50 years, it is not surprising that the consequent disability has also increased [14,24]. High BMI continues to be an important risk factor for musculoskeletal disorders, and the World Health Organization’s approach, which is to prevent and control high BMI as a stand-alone disease rather than controlling the sequelae of high BMI, would be our recommendation [25].

The present study assesses the burden of osteoarthritis in Europe, with a particular focus on its age-related distribution. Additionally, we provide up-to-date estimates of the burden of disability associated with osteoarthritis in individuals under 50 years, highlighting its impact on the labor force. Our study, however, was limited by its focus on sub-regional differences in the burden of osteoarthritis and did not discuss national and sub-national estimates. Additionally, the study does not discuss the burden of osteoarthritis based on various anatomical regions, such as the hip and knee. Finally, according to the GBD 2021 database, no osteoarthritis burden has been recorded for individuals aged 15-29 years, whereas burden estimates have been assigned to those aged 30-49 years. Consequently, the aggregated burden for the 15-49 years age group may be underestimated, as population data for the 15-29 years cohort were incorporated into the calculations despite the absence of attributed burden within that subgroup.

## Conclusions

We identified an overall increase in the burden of osteoarthritis in Central, Eastern, and Western Europe between 1990 and 2021. Western Europe had the highest incidence, prevalence, and disability rates in young adults and all ages. Western Europe also had the highest burden of osteoarthritis attributable to high BMI in individuals aged under 50 years and all ages. Country-level data revealed a higher incidence of osteoarthritis among females in all countries within the region. Central Europe recorded a 70% rise in osteoarthritis-related disability, and Eastern Europe recorded an 82.5% rise in osteoarthritis disability in young people attributable to high BMI. We believe that these findings provide policy-relevant insights by quantifying the changes in disability attributable to high BMI in the population and in individuals aged under 50 years.
